# Natural History, Recurrence, and Progression in Superficial Bladder Cancer

**DOI:** 10.1100/tsw.2006.404

**Published:** 2006-03-27

**Authors:** Richard J. Sylvester

**Affiliations:** European Organisation for Research and Treatment of Cancer Data Center, 83 avenue E Mounier, 1200 Brussels, Belgium

**Keywords:** bladder cancer, natural history, prognostic factors, recurrence, progression

## Abstract

Superficial bladder cancer encompasses patients with stage Ta T1 tumors and patients with carcinoma *in situ* (CIS). The natural history or treatment-related prognosis of these patients varies considerably from one patient to the next based on the patients clinical and the tumor's pathological characteristics. Based on a review of the literature, the most important prognostic factors for recurrence are the prior recurrence rate, number of tumors, and tumor size; whereas for progression, the most important prognostic factors are the T category, grade, and presence of CIS. Treatment with intravesical bacillus Calmette-Guerin reduces both the risk of recurrence and the risk of progression, and is the treatment of choice in high-risk papillary tumors and in patients with CIS. Assessment of a patient's prognostic factors and his or her risk of recurrence and progression is a prerequisite for determining the most appropriate treatment and frequency of follow-up for a given patient.

## INTRODUCTION

Bladder cancer is a multifocal disease that may pass through different stages of development, from atypia to dysplasia to overt tumor due to the cumulative exposure to a variety of potential carcinogens and the continual onslaught of the carcinogenic process. The term "superficial bladder cancer" encompasses a quite heterogeneous group of patients. As defined in the $6^{th}$ TNM Classification of Malignant Tumors[1], superficial bladder cancer includes stage Ta (confinement to the epithelium or mucosa) and T1 (invasion of the subepithelial connective tissue or lamina propria) papillary tumors, irrespective of the grade, and carcinoma *in situ* (CIS)(Tis: flat, high-grade, nonpapillary carcinomas confined to the urothelium). It has been estimated that 70% of all superficial bladder cancer patients have Ta tumors, of which approximately 7% are grade 3 according to the 1973 WHO classification system, and that between 5 and 10% have CIS[2,3,4,5].

Taken together as a group, patients with superficial bladder cancer have a median survival of approximately 10 years, however, the vast majority of deaths are not due to bladder cancer[6]. Their natural history is characterized by the disease's propensity to recur as a superficial tumor and to progress to muscle-invasive disease, stage T2 or higher, which is associated with a much higher risk of death due to bladder cancer[7]. Despite having a long median survival, grouping all these patients together under the umbrella "superficial" bladder cancer is misleading since, as described below, one patient's prognosis can be quite different than that of another patient.

When considering a patient's natural history or prognosis, it is necessary to consider not only his or her clinical and pathological factors, one must also take into account the potential effect of the intravesical treatment received: chemotherapy or immunotherapy.

## CLINICAL AND PATHOLOGICAL PROGNOSTIC FACTORS

With respect to the "untreated" natural history of patients with superficial bladder cancer, the most important distinction is between patients with only stage Ta T1 transitional cell carcinoma and patients with CIS. In 906 stage Ta T1 bladder cancer patients without CIS who did not initially receive any intravesical treatment after transurethral resection (TUR), 47% of the patients recurred and 9% progressed to muscle-invasive disease based on a median follow-up of about 5 years. Approximately one-third of the patients died and one-third of the deaths were due to malignant disease[6]. On the other hand, CIS, which cannot be eradicated by TUR alone, has a much higher progression rate than most Ta and T1 tumors. Based on 14 series of patients treated with TUR alone, mostly in the 1970s prior to the widespread use of intravesical treatment, Lamm found that 54% of 382 patients with CIS progressed to muscle-invasive disease[8].

Prognostic factors in patients with superficial bladder cancer have been the subject of numerous publications over the past 20 years. In most cases, they have been based on patients who received intravesical chemotherapy, either prior to or after the first recurrence. However, publications do not always agree concerning the prognostic importance of the different factors. Discrepancies may result due to differences in the number of patients included in the analysis, the treatment they received, the choice and definition of the endpoint, the duration of follow-up, the variables to be analyzed and their coding, whether the analysis is univariate or multivariate, and, in this last case, the model used and how it is fit, and correlations between the different factors entered in the model.

Tables 1 and 2 summarize the results of 19 publications that assessed the prognostic importance of some of the more common clinical and pathological factors that have been studied[9,10,11,12,13,14,15,16,17,18,19,20,21,22,23,24,25,26,27]. Based on multivariate analyses, the most frequently cited prognostic factors for time to first recurrence are the number of tumors, whether the patient is primary or recurrent, and, if recurrent, the prior recurrence rate and the tumor's size (Table 1). For the time to progression to muscle-invasive disease, the most important prognostic factors are the grade, stage (T category), and the presence of CIS (Table 2). These studies have shown that depending on a patient's characteristics, the probability of recurrence at 1 year varies between approximately 15 and 70%[9] while the probability of progression at 5 years ranges between about 7 and 40%[10]. Cytology, whose sensitivity is correlated with tumor grade, has also been found to be of prognostic importance in a number of studies.

As previously indicated, most of these prognostic factor analyses have been carried out in patients treated with intravesical chemotherapy. Few large-scale prognostic factor studies exist in patients treated with intravesical bacillus Calmette-Guerin (BCG). In a multivariate analysis of 221 patients treated with BCG, 62% of whom did not receive maintenance treatment, Herr et al.[19] found that tumor multifocality, stage, positive cytology, and previous duration of bladder cancer were related to progression. In a more recent series from Herr and Dalbagni, in 93 patients treated with BCG, the response to BCG at 6 months was the most important factor for subsequent tumor recurrence and progression, independent of whether or not patients received maintenance[28]. In 26 patients with primary CIS, Orsola et al.[29] also found response to BCG at 6 months to be an important prognostic factor for subsequent progression. More generally, Saint et al.[30] provide an overview of host and immunological characteristics; histopathologic, cytologic, endoscopic, and molecular tumor characteristics; and histopathologic characteristics after BCG

**TABLE 1**
**Prognostic Factors for Recurrence**

| | Univariate Analysis | Multivariate Analysis |
|---|---|---|
| Age | Shariat[24] | Shariat[24] |
| Gender | — | — |
| Prior recurrence rate | Ali-el-Dein[11]<br>Dalesio[18]<br>Kaasinen[21]<br>Kurth[10]<br>Sylvester[27] | Ali-el-Dein[11]<br>Dalesio[18]<br>Kaasinen[21]<br>Kurth[10]<br>Sylvester[27] |
| Number of tumors | Ali-el-Dein[11]<br>Allard[9]<br>Dalesio[18]<br>Holmang[20]<br>Kaasinen[21]<br>Kurth[10]<br>Millan-Rodriguez[15,16]<br>Parmar[14]<br>Sylvester[27] | Ali-el-Dein[11]<br>Allard[9]<br>Dalesio[18]<br>Kaasinen[21]<br>Kiemeney[13]<br>Millan-Rodriguez[15,16]<br>Parmar[14]<br>Sylvester[27] |
| Tumor site | Kurth[10]<br>Parmar[14] | Kurth[10] |
| Tumor size | Allard[9]<br>Millan-Rodriguez[15,16]<br>Parmar[14]<br>Sylvester[27] | Allard[9]<br>Dalesio[18]<br>Kurth[10]<br>Millan-Rodriguez[15,16]<br>Sylvester[27] |
| T category | Ali-el-Dein[11]<br>Allard[9]<br>Holmang[20]<br>Parmar[14]<br>Sylvester[27] | Ali-el-Dein[11]<br>Allard[9]<br>Kiemeney[13]<br>Sylvester[27] |
| Grade | Allard[9]<br>Dalesio[18]<br>Holmang[20]<br>Kurth[10]<br>Millan-Rodriguez[15,16]<br>Parmar[14]<br>Sylvester[27] | Allard[9]<br>Holmang[20]<br>Kurth[10]<br>Sylvester[27] |
| Configuration: Papillary vs. nonpapillary | Ali-el-Dein[11] | Ali-el-Dein[11] |
| CIS | Sylvester[27] | Millan-Rodriguez[15,16] |
| Recurrence at 3 months | Ali-el-Dein[11]<br>Holmang[20]<br>Kurth[10]<br>Parmar[14] | Ali-el-Dein[11]<br>Holmang[20]<br>Kurth[10]<br>Parmar[14] |

that are related to recurrence and progression in patients treated with BCG. Except for the patient's response to BCG at 3 or 6 months, they conclude that no definitive prognostic factors have been identified thus far. It is thus still unknown if the factors identified in Tables 1 and 2 are of similar prognostic importance in patients treated with chemotherapy and in patients treated with BCG, especially when maintenance is used.

In patients with T1 tumors, the depth of invasion, grade, and the presence of concomitant CIS have all been identified as important prognostic factors for progression. The depth of lamina propria invasion, i.e., whether the tumor is superficial to, into, or beyond the muscularis mucosae (T1a, T1b, T1c), has been linked to the risk of progression to muscle-invasive disease in several studies, even in BCG-treated patients[17,23,25].

**TABLE 2**
**Prognostic Factors for Progression**

| | Univariate Analysis | Multivariate Analysis |
|---|---|---|
| Age | Kurth[10]<br>Shariat[24]<br>Sylvester[27] | Shariat[24] |
| Gender | — | Kurth[10] |
| Prior recurrence rate | Herr[19]<br>Kurth[10]<br>Sylvester[27] | Herr[19]<br>Kurth[10]<br>Sylvester[27] |
| Number of tumors | Fujii[12]<br>Millan-Rodriguez[15,16]<br>Sylvester[27] | Kiemeney[13]<br>Millan-Rodriguez[15,16]<br>Sylvester[27] |
| Tumor site | Fujii[12]<br>Kurth[10] | Fujii[12]<br>Kurth[10] |
| Tumor size | Kurth[10]<br>Millan-Rodriguez[15,16]<br>Sylvester[27] | Kurth[10]<br>Millan-Rodriguez[15,16]<br>Sylvester[27] |
| T category | Bernardini[17]<br>Fujii[12]<br>Herr[19]<br>Holmang[20]<br>Kurth[10]<br>Millan-Rodriguez[15,16]<br>Orsola[23]<br>Smits[25]<br>Sylvester[27] | Bernardini[17]<br>Fujii[12]<br>Herr[19]<br>Kiemeney[13]<br>Orsola[23]<br>Sylvester[27] |
| Grade | Ali-el-Dein[11]<br>Bernardini[17]<br>Fujii[12]<br>Herr[19]<br>Holmang[20]<br>Kurth[10]<br>Millan-Rodriguez[15,16]<br>Sylvester[27] | Ali-el-Dein[11]<br>Fujii[12]<br>Holmang[20]<br>Kiemeney[13]<br>Kurth[10]<br>Millan-Rodriguez[15,16]<br>Sylvester[27] |
| Configuration: papillary vs. nonpapillary | Ali-el-Dein[11]<br>Fujii[12] | — |
| CIS | Bernardini[17]<br>Millan-Rodriguez[15,16]<br>Orsola[23]<br>Smits[25]<br>Sylvester[27] | Bernardini[17]<br>Millan-Rodriguez[15,16]<br>Orsola[23]<br>Sylvester[27] |
| Recurrence at 3 months | Ali-el-Dein[11]<br>Holmang[20]<br>Kurth[10]<br>Lockyer[22]<br>Solsona[26]<br>Sylvester[27] | Ali-el-Dein[11]<br>Holmang[20]<br>Kurth[10]<br>Solsona[26]<br>Sylvester[27] |

Orsola et al.[23] found that the progression rate for deep lamina propria–invasive tumors (T1b and T1c) was 34% as compared to 8% for T1a tumors.

The poor prognosis of patients with T1 G3 tumors has also been the subject of various publications with ongoing debates over the most appropriate treatment, i.e., cystectomy or conservative treatment with intravesical BCG[31,32,33]. Some authors have suggested, however, that the prognosis of T1 G3 patients is not uniform, but that the risk of progression depends on the patient's other characteristics, in particular the presence of concomitant CIS, which confers a particularly poor prognosis[23,27,31,32,33,34]. Orsola et al. found that CIS was associated with more than 50% of high-grade T1 tumors that progressed[23]. In a series of 194 patients with T1 G3 tumors, 74% of patients with concomitant CIS progressed by 5 years as compared to 29% of patients without concomitant CIS[27].

Once CIS is diagnosed, there are no completely reliable prognostic factors that can be used to predict the course of the disease that can be quite variable. Since most publications on CIS are based on relatively small numbers of patients, there is still considerable uncertainty concerning the exact importance of various factors. The most frequently cited prognostic factors are age, response to BCG, type of CIS (primary, secondary, concurrent), extent of CIS (unifocal, multifocal, or diffuse), irritative bladder symptoms, hematuria, and extravesical extension (prostatic urethra, upper tract)[5].

In patients with CIS, the response to BCG at 3 and 6 months after starting treatment is one of the most important factors for the future course of the disease. Approximately 10–20% of complete responders will eventually progress as compared to two-thirds or more of the nonresponders. Patients with primary CIS (isolated CIS with no history of papillary tumors) have the best long-term prognosis, while those with both CIS and papillary tumors (concurrent CIS) have the worst prognosis[5].

Molecular markers such as p53, Ki-67, NMP22, and Cox-2, measured pre- and/or post-treatment, have all been shown to be of some promise; however, they have not been sufficiently validated to be used to predict a patient's prognosis on a day-to-day basis at this time[17,24,34,35,36].

Recurrence at the first follow-up cystoscopy has been identified to be one of the most important prognostic factors for both future recurrence and progression to muscle-invasive disease[10,11,14,20, 22,26,27]. In 74 patients with stage T1 tumors who received either intravesical chemotherapy or BCG, Holmang and Johansson[20] found that 18% of patients with a negative first cystoscopy progressed as compared to 41% of patients with a positive first cystoscopy. In another group of 111 patients with CIS and 80 patients with high-grade T1 disease, the response at 3 months was the most important prognostic factor for subsequent progression: 11% of complete responders progressed as compared to 66% of nonresponders[26].

In a series of 316 patients with a recurrence at the first follow-up cystoscopy, 209 patients (66%) had a recurrence at the same site as initially involved and 166 patients (53%) had a recurrence at a different site[37]. A wide variation between institutions in the recurrence rate at the first follow-up cystoscopy was also noted, both for patients with single tumors and patients with multiple tumors[37]. Recurrence at the first follow-up cystoscopy may be due to residual tumor that was incompletely resected, tumor that was present but not visible (or overlooked) at the initial TUR, tumor cell implantation at the site of resection, or aggressive tumor biology. However, these results are all based on studies done prior to the use of one immediate instillation of chemotherapy[38] and a second-look TUR approximately 4 weeks after the initial TUR[39]. Nowadays, with the more widespread use of one immediate instillation of chemotherapy and second-look TUR, the percentage of patients with recurrence at 3 months should be less, but those who recur are likely to have a very poor prognosis.

One of the main goals of prognostic factor analyses has been to divide patients into risk groups of good, intermediate, and poor prognosis in order to adapt the treatment according to the risk group[9,10,11,12,13,14,15,40]. When the various factors in the multivariate model are of approximately the same importance, a simplified prognostic scoring system based on the number of adverse tumor characteristics can be used to classify patients into different risk groups[9].

In some cases, the same risk group classification has been applied to both recurrence and progression even though the relative importance of the prognostic factors for these two endpoints is different[15,16]. A patient that is at high risk of recurrence may be at low risk for progression, so the definition of the risk groups should be endpoint dependent. Thus, a risk group classification may be appropriate for one endpoint, but not for another. In addition, the division of patients into risk groups is arbitrary since the concept of what constitutes a good-risk patient or a poor-risk patient may vary from one clinician to another.

Nomograms have been proposed as a method that avoids the arbitrary division of patients into risk groups[24]. Based on a patient's prognostic factors, one can calculate the probability of a certain event within a given period of time; for example, the probability of tumor recurrence within the 1st year or the probability of progressing to muscle-invasive disease within 5 years. Alternatively, look-up tables provide such probabilities based on the prognostic score. This last approach was adopted by the European Organisation for Research and Treatment of Cancer (EORTC), which has developed risk tables that can be used to predict an individual patient's probability of recurrence and progression based on six routinely assessed factors: prior recurrence rate, number of tumors, tumor size, T category, grade, and concomitant CIS[27]. Based on these tables, the probability of recurrence varied from 15–61% at 1 year and from 31–78% at 5 years. The probability of progression varied from 0.2–17% at 1 year and from 0.8–45% at 5 years, again showing that the prognosis of patients labeled as having "superficial" bladder cancer is quite heterogeneous. With these probabilities, the urologist can discuss the different treatment options with the patient in order to determine the most appropriate treatment and frequency of follow-up. Electronic versions of the risk table calculators for Windows 2000 and XP, Windows Handheld and Palm devices are available at www.eortc.be/tools/bladdercalculator.

## INTRAVESICAL TREATMENT

Trials assessing different intravesical chemotherapeutic agents such as thiotepa, doxorubicin, epirubicin, and mitomycin C have been carried out in an attempt to find treatments that, after TUR, reduce the recurrence rate and delay or prevent progression to muscle-invasive disease.

A meta-analysis of 6 studies with 2535 patients has shown that intravesical chemotherapy prolongs the time to first recurrence. It reduced the risk of recurrence by 20% and increased the 8-year disease-free rate from 37–45%[6]. However, intravesical chemotherapy had no effect on delaying or preventing progression to muscle-invasive disease.

One immediate post-TUR instillation of chemotherapy has been particularly effective in reducing the percentage of patients who recur after TUR, from 48–37% in a recent meta-analysis. Both patients with a single tumor and patients with multiple tumors benefited from one immediate instillation; however, it was shown that one instillation by itself is insufficient treatment in patients with multiple tumors[38]. A second-look TUR was not included in these studies as they all began more than 10 years ago and included mostly good-risk patients.

Intravesical BCG has been proposed as an alternative to intravesical chemotherapy, especially in patients at high risk of progression where intravesical chemotherapy has not been shown to be effective. As compared to intravesical mitomycin C, a meta-analysis has shown that BCG reduces the percentage of patients who recur, from 46–38% overall, and from 54–37% in studies where maintenance BCG was given[41]. Other meta-analyses have shown that BCG with maintenance also reduces the risk of progression to muscle-invasive disease. Compared to mitomycin C, maintenance BCG reduced the percentage of patients who progressed to muscle-invasive disease from 14–10%, a reduction of 34% in the risk of progression[42]. In a second meta-analysis including other alternative treatments, maintenance BCG reduced the percentage of patients who progressed from 15–10%, a reduction of 37% in the risk of progression[43]. Separate results according to disease stage (Ta, T1) and grade were not available.

In a meta-analysis of 700 patients with CIS, intravesical treatment with BCG has likewise been found to be more effective than treatment with intravesical chemotherapy[44]. Intravesical BCG increased the complete response rate from 51–68%, decreased the percentage of complete responders who recurred from 50–34%, and based on a median follow up of 3.6 years, increased the long-term percentage of patients who remained disease free from 26–47%. BCG appeared to be superior to mitomycin C only in the trials where maintenance BCG was given. The median duration of complete response with maintenance BCG is approximately 5 years.

BCG thus favorably influences the natural history and prognosis of patients with superficial bladder cancer. However, as intravesical BCG is associated with more local and systemic side effects than intravesical chemotherapy, chemotherapy has generally been considered to be the treatment of choice in good prognosis patients who are unlikely to progress and where the goal is to prevent or reduce recurrences. On the other hand, BCG is the treatment of choice in patients at high risk of recurrence and especially in those patients who are at high risk of progression.

## CONCLUSIONS

Prognostic factor studies have thus shown that patients labeled as having superficial bladder cancer have, in fact, a quite heterogeneous prognosis. Patients with a primary, single, small Ta G1 tumor without CIS have a very low chance of progression, less than 1%, while patients with T1 G3 tumors and concomitant CIS have a much higher risk of progression, approaching 70% or more.

However, a limitation of nearly all these prognostic factor studies is that they date from the pre-BCG maintenance era, most patients did not receive an immediate postoperative instillation of chemotherapy, and high-risk patients did not have a second-look TUR. Thus, current rates of recurrence and progression may be lower than those previously reported in the literature. Likewise, it is not clear if the prognostic importance of factors identified in previous studies would remain the same if patients are treated according to current recommendations: the use of an immediate instillation, and a second look TUR and maintenance BCG in high-risk patients.

Nevertheless, it is time to stop classifying all these patients under the same heading, superficial, in order to properly convey the very different risk of progression to muscle invasive disease that is present in these patients[45,46]. Nieder and Soloway[46] propose a new nomenclature based on the pathological diagnosis and the likelihood of progression:

- Papilloma, papillary urothelial neoplasms of low malignant potential (PUNLMP)
- Ta, low grade
- Ta, high grade
- T1, high grade
- CIS

In this way, both the treatment and frequency of follow-up can be better adapted according to the patient's prognosis.

## ACKNOWLEDGMENTS

This research project was supported by grant number 5U10 CA11488-35 from the National Cancer Institute (Bethesda, MD). The contents of this publication are solely the responsibility of the authors and do not necessarily represent the official views of the National Cancer Institute. The EORTC is grateful to the Fondation contre le Cancer (Belgium) for also providing support for this research project.
